# Maximal Load of the Vitamin B12 Transport System: A Study on Mice Treated for Four Weeks with High-Dose Vitamin B12 or Cobinamide

**DOI:** 10.1371/journal.pone.0046657

**Published:** 2012-10-01

**Authors:** Dorte L. Lildballe, Elena Mutti, Henrik Birn, Ebba Nexo

**Affiliations:** 1 Department of Clinical Biochemistry, Aarhus University Hospital, Aarhus, Denmark; 2 Department of Clinical Genetics, Aarhus University Hospital, Aarhus, Denmark; 3 Departments of Nephrology, Aarhus University Hospital and Biomedicine, Aarhus University, Aarhus, Denmark; Stem Cell Research Institute, Belgium

## Abstract

Several studies suggest that the vitamin B12 (B12) transport system can be used for the cellular delivery of B12-conjugated drugs, also in long-term treatment Whether this strategy will affect the endogenous metabolism of B12 is not known. To study the effect of treatment with excess B12 or an inert derivative, we established a mouse model using implanted osmotic minipumps to deliver saline, cobinamide (Cbi) (4.25 nmol/h), or B12 (1.75 nmol/h) for 27 days (n = 7 in each group). B12 content and markers of B12 metabolism were analysed in plasma, urine, kidney, liver, and salivary glands. Both Cbi and B12 treatment saturated the transcobalamin protein in mouse plasma. Cbi decreased the content of B12 in tissues to 33–50% of the level in control animals but did not influence any of the markers examined. B12 treatment increased the tissue B12 level up to 350%. In addition, the transcript levels for methylenetetrahydrofolate reductase in kidneys and for transcobalamin and transcobalamin receptor in the salivary glands were reduced. Our study confirms the feasibility of delivering drugs through the B12 transport system but emphasises that B12 status should be monitored because there is a risk of decreasing the transport of endogenous B12. This risk may lead to B12 deficiency during prolonged treatment.

## Introduction

**Figure 1:**
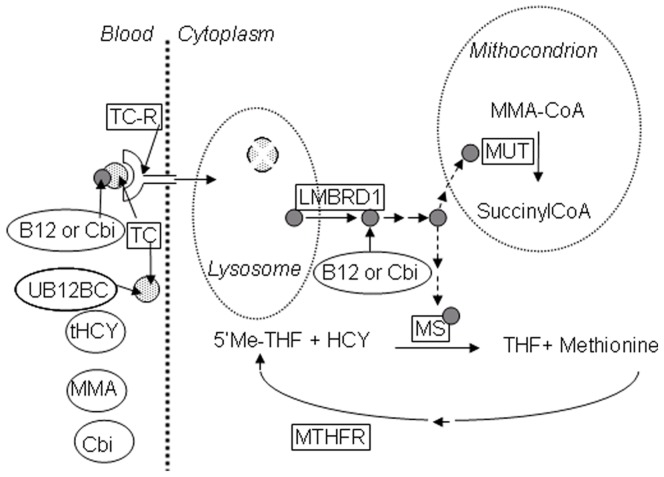
Simplified overview of B12 metabolism. A simplified overview of vitamin B12 (B12) metabolism in mice with focus on the analysed parameters. Through binding to the transcobalamin receptor (TC–R), the TC–B12/cobinamide(Cbi) complex is internalised into the lysosomes. TC is degraded and B12 is transported into the cytoplasm by the lysosomal membrane transporter 1 (LMBRD1). Intracellular, B12 serves as cofactor for the mitochondrial methylmalonylCoA mutase (MUT) and the cytosolic methionine synthase (MS) that acts in coordination with methylenetetrahydrofolate reductase (MTHFR). In plasma, TC circulates partly unsaturated with B12 (UB12BC). If the B12 supply to the cell is insufficient, methylmalonic acid (MMA) and homocysteine (tHcy) accumulates in the blood. The transcript level of the boxed components were analysed by quantitative reverse–transcript (q–rt–) PCR and the circularised components were analysed biochemically in either tissue or blood (plasma) as indicated.

Once absorbed from the intestine, vitamin B12 (B12) is transported to all cells to play its role as cofactor for B12 dependent enzymes. These processes imply a coordinated action of several proteins and receptors, as outlined in Figure 1 (for a resent review, see [1]). The plasma carrier protein, transcobalamin (TC) plays a key role for cellular uptake of B12. TC is the only B12 binding protein present in mouse plasma [2] while humans express the additional plasma transporter, haptocorrin (HC), a protein of unknown function [3]. In humans, TC and HC recognize different forms of B12. Human TC only binds the active forms of B12 while HC also binds B12 analogues such as cobinamide (Cbi) [4]. Mouse TC have features common to both human TC and HC, since it promotes cellular uptake of B12 but at the same time mouse TC recognizes both B12 and Cbi [2].

Through binding to the TC receptor (TC-R, CD320), the TC-B12 complex is internalised into the lysosomes as illustrated in Figure 1. Inside the lysosome, TC is degraded and the liberated B12 is transported into the cytoplasm by the lysosomal membrane transporter 1 (LMBRD1). Intracellular B12 serves as cofactor for the mitochondrial methylmalonyl-CoA mutase (MUT) and the cytosolic methionine synthase (MS) that acts in coordination with methylenetetrahydrofolate reductase (MTHFR) for the conversion of homocysteine (HCY) to methionine. B12 depletion will cause an increase in the metabolites methylmalonic acid (MMA) and HCY as their enzymatic conversion are reduced. The consequences of B12 deficiency in humans include megaloblastic anaemia and neurological abnormalities [1], while the consequence in model animals such as rodents to our knowledge is unsettled.

Several researchers have suggested use of the B12 transport system to deliver B12-conjugated drugs and nano particles into cells [5]–[8], especially in the case of life-long treatments such as those for diabetic patients. This strategy may lead to a B12 overload of the cells if the B12-conjugate is cleaved to form conjugate and metabolic active B12 inside the cell or to interference with B12 metabolism if this is not the case. However, at present we do not know to what extent an overload with active or inactive B12 influences the B12 metabolism.

Since mouse TC recognises both B12 and the biologically inert B12 analogue Cbi [2]. we were able to study an overload of the B12 transport system induced by B12 or Cbi. We report that infusion of Cbi leads to depletion of B12 from mice tissues and that overload with B12 leads to alterations in the metabolism of HCY.

## Materials and Methods

### Ethics statement

All animal experiments were carried out in accordance with provisions for the animal care license provided by the Danish National Animal Experiments Inspectorate. The protocol was approved by the Danish National Animal Experiments Inspectorate (provision no 2010/561–1855). Surgery was performed under isoflurane anaesthesia, and pain after surgery was reduced as described below.

### Animals

21 7-week-old female mice (strain 129.S6; Taconic, Denmark) were divided into three groups nd caged separately: control mice (C) n = 7 cobinamide-loaded mice (Cbi) n = 7; and vitamin B12-loaded mice (B12) n = 7. The mice had free access to water and to standard chow (Altromin maintenance diet for rats and mice (1324) (19 pmol/g B12; 0.4 pmol/g Cbi) Altromin, Germany) except during 24-h urine collection once weekly in metabolic cages. After 1 week of acclimation (age 8 weeks), an osmotic minipump (Mini-Osmotic Pump Model 2004, Alzet Cupertino, CA, USA) was implanted into each animal. The mice were anesthetised with isoflurane (IsoFlo® Vet), Abbott), and osmotic minipumps were inserted subcutaneously into the back by incision just below the neck region. After insertion, the wound was closed by absorbable suture. Prior to insertion, the pumps were equilibrated and filled following the manufacturer's instructions. The pumps were filled with either saline (0.9% NaCl) (control mice), 17 mM dicyano-cobinamide (MW: 1042.12 g/mol Sigma-Aldrich, Saint Louis, MO, USA) in 0.9% NaCl (Cbi mice), or 7 mM cyanocobalamin (MW: 1355.37 g/mol; Sigma-Aldrich) in 0.9% NaCl (B12 mice). The latter represented the maximal amount of B12 that could be dissolved. According to the manufactures, the delivery rate of the pumps is 0.25 µL/hr equivalent to delivered rate of 4.25 nmol/h for Cbi mice and 1.75 nmol/h for B12 mice. To avoid wound biting between mice, the mice were housed in individual cages for 3 days after surgery. In addition, to ameliorate pain after surgery, analgesics was put into the drinking water (buprenorphine hydrochloride 0.06 mg/ml) for 3 days post operation. 27 days after insertion of the osmotic minipumps, mice were anaesthetised using isoflurane. Following, they were sacrificed by exsanguination followed by cutting of the thorax and heart.

### Urine and blood collection

The mice were weighed just after insertion of the pumps (day 1) and on days 5, 12, 19, and 27. Prior to urine collection, each mouse received an intraperitoneal injection of 250 µL 0.9% NaCl to increase urine output and were placed in individual, metabolic cages for 24 h with free access to water. 1 µL 20% Na-azide was added to each urine collection tube to prevent bacterial growth.

Following urine collection, blood was sampled from the sublingual vein (∼50 µL total volume) [9]. Blood samples were heparinised, centrifuged at 4,000 g for 8 min at room temperature, and the plasma fraction was collected. Urine and plasma were stored at −20°C.

On the day of sacrifice (day 27), mice were anaesthetised with isoflurane and blood was collected from the inferior caval vein. One aliquot (∼200 µL) of blood from each mouse was transferred to a dry EDTA tube for haematological analysis. The remaining blood was collected in heparinised tubes, and plasma was prepared as described above.

### Organ collection

Immediately after the mice were sacrificed, organs were collected and snap-frozen in liquid nitrogen. The organs were stored at −80°C until further processing.

### Crude tissue extraction

Crude aliquots of tissue extracts were prepared in homogenisation buffer containing protease inhibitors as described previously [2]. The tissue-to-buffer ratios were: 231 g/L (kidney); 400 g/L (liver), and 140 g/L (salivary glands (submaxillary glands)).

### Analysis of haematological values

Within 2 hours after collection, 200 µL of EDTA blood from each mouse was analysed for haematological parameters on a Sysmex XE-2100 Automated Hematology System (Sysmex Corporation) [10].

### MMA, tHcy, cysteine, and methionine in plasma

50 µL of heparinised plasma collected on day 27 was sent to BeVital (http://www.bevital.no/) for analysis of methylmalonic acid (MMA), total homocysteine (tHcy), total cysteine, and methionine levels using standardised GC-MS methods.

### MMA in urine

MMA in mice urine was measured using LC-MSMS (1290 LC-system and 6490 MS-detector; Agilent) [11].

### Unsaturated B12 binding capacity (UB12BC)

Unsaturated B12 binding capacity (UB12BC), representing the amount of B12-binding-protein not bound to B12 at the time of sampling, was carried out as described previously [12].

### B12 in urine

B12 levels were measured in 24-h urine by a competitive electrochemiluminescence immunoassay on a Cobas 6000e immunoassay system using the analytical kit supplied by the manufacturer (Roche Diagnostics) [13]. Where requested, the samples were diluted with 0.9% NaCl prior to analysis.

### ELISA-based analysis of B12 and Cbi

The corrinoids, B12 and B12-like compounds, were extracted from their carrier proteins by acidic denaturation and boiling [14]. Briefly, 5 µL of crude protein extract (liver, kidney, salivary glands), plasma, or urine were mixed with 95 µL 0.9% NaCl and 400 µL 0.3 M Na-acetate at pH 4.8 with 75 µM KCN and incubated at 100°C for 15 min followed by cooling and centrifugation for 15 min at 16,000 g. 250 µL of supernatant was mixed with 25 µL 2 M Tris-HCl buffer pH 8.5. The concentration of total B12 and total corrinoids was analysed using an ELISA-based method employing human transcobalamin for the measurement of B12 and human haptocorrin for the measurement of total corrinoids, as described previously [15]. The concentrations of analogues were calculated as the differences between total corrinoids and B12.

### Analysis of gene transcription level

#### RNA extraction and cDNA synthesis

Total RNA was extracted from tissue aliquots (20–50 mg) using a QIAamp RNA kit and QIAcube instrument according to the instructions of the manufacturer (Qiagen, Merck Eurolab A/S). All samples were DNase-treated using the RNase-Free DNase Set according to the instructions of the manufacturer (Qiagen, Merck Eurolab A/S).

The RNA concentration in each extract was determined by optical density at wavelengths of 260 nm by use of a GeneQuant II (Pharmacia Biotech, Cambridge, UK). cDNA was synthesised as described previously [2] and stored at −20°C until analysed.

#### Quantitative reverse transcriptase PCR

Quantitative (q) reverse transcriptase (rt) PCR was carried out using real time PCR, as described previously [2]. Calibration, positive, and negative samples were included in each run. The calibration curve was composed of serial dilutions of cDNA from a pool of liver control mice. Data on primer sequences and annealing temperatures are given in Table 1.

**Table 1:** Primer pairs used in quantitative reverse-transcriptase PCR on mouse tissue.

Protein name	Acc. No	Forward primer (pmol/µL)	Reverse primer (pmol/µL)	T^1^ (°C)
Methionine synthase (MS)	NM_001081128	5’-GCAGATGTGGCCAGAAAAG-3’ (5) [23]	5’-GCCACAAACCTCTTGACTC-3’ (5) [23]	60
Methylenetetrahydrofolate reductase (MTHFR)	NM_010840	5’-AGCTTGAATCCACCTGGACTGTAT-3’ (5) [23]	5’-AGACTAGCGTTGCTGGTTTCAGA-3’ (5) [23]	56
Methylmalonyl-Coenzyme A mutase (MUT)	NM_008650.3	5’-GCAGGCTTTAGTACTGTGG -3’ (10)	5’-CCAAGTCAAAGGCAACAGAC-3’ (10)	60
Lysosomal cobalamin transporter (LMBRD1)	NM_026719	5’-CTGGAGAACACGGAGGACAT-3’ (10)	5’-GCTTTAAGGCACGTCTATCC-3’ (10)	56
Transcolabamin (TC)	NM_015749	5’-GATGTCCTGAAGTTGGCACA-3’ (5) [2](5)	5’-TCCTGGGGTTTGTAGTCAGC-3’ (5) [2] (5)	60
TC-receptor (TC-R)	NM_19421.3	5’-GACTGCTCTGATGGCAGTGA-3’ (5) [2] (5)	5’-GCCACGTGTGTGGAATACAG-3’ (5)[2] (5)	60
β-Actin	NM_007393.3	5’- AGGTGACAGCATTGCTTCTG -3’	5’-GCTGCCTCAACACCTCAAC-3’	60

1: Annealing temperatures used in q-rt-PCR for the given primer pair.

The fluorescence data were collected automatically, and the mRNA levels were quantified using LightCycler 480 (Roche A/S) software version 3.3 with the second derivative maximum method of quantification.

For each gene, the results were obtained as absolute values relative to the mRNA content in the calibration curve. The results obtained are normalised against the concentration of β-actin mRNA in the same sample. To ease the comparison of control to treated mice, we normalised against the transcript level in control mice, setting the mean transcript concentration of control mice to 100.

## Data Analysis

Data are presented as median (range). The differences in various biochemical markers between groups were tested by one-way analysis of variance (ANOVA). Non-parametric Wilcoxon tests were used to compare data of q-rt-PCR [16]. The calculations were done using Microsoft Office Excel 2003 and Graph Pad Prism 4. A p<0.05 was considered significant.

## Results

### B12 and its related parameters in normal mice

The plasma levels of haematological and biochemical markers as well as the concentration of B12 and B12 analogues in plasma, kidney, liver, salivary gland, and urine of control mice are shown in Table 2. This is, to our knowledge, the first extensive overview of markers related to B12 status measured in the same group of mice. Consistent with previous data [2], we report that kidney tissue contains the highest concentration of B12 in normal mice.

**Table 2:** B12-related parameters measure in mice following 27 days of continuous delivery of saline (control), cobinamide (Cbi) and vitamin B12 (B12) by osmotic minipumps^1^.

	ControlMedian (range)	Cbi^2^Median (range)	B12^2^Median (range)
**Haematological parameters in blood**			
White blood cell count (10^−9^L)	1.7 (1.4 – 2.6)	2.6 (1.1 − 3.3)	3.6 (2.04 − 6.0)^*^
Red blood cell count (10^−12^L)	9.4 (9.0 – 9.7)	9.2 (9.1 – 9.5)	9.6 (9.3 – 9.9)
Haemoglobin (mM)	9.6 (9.2 – 9.9)	9.4 (9.0 – 9.9)	9.8 (9.6 – 9.9)
Erythrocyte volume fraction (ratio)	0.50 (0.49 – 0.52)	0.49 (0.47 – 0.51)	0.50 (0.50 – 0.52)
Erythrocyte mean cell volume (fL)	53 (53 – 54)	53 (52 – 54)	53 (52 – 54)
Erythrocyte mean cell haemoglobin concentration (mmol/L)	19 (19 – 20)	19 (19 – 20)	19 (19 – 20)
**B12, Cbi and related biochemical parameters in plasma**			
B12 (nmol/L)	25 (21 – 30)	19 (16 – 23)^*^	160 (120 – 240)^***,###^
Cbi (nmol/L)	<0.9	820 (650 – 1100)^***^	<0.9^###^
UB12BC (nmol/L)	28.4 (23.8 – 31.8)	<2^***^	<2^***,###^
MMA (µmol/L)	0.45 (0.29 – 0.62)	0.50 (0.28 – 0.67)	0.33 (0.23 – 0.42)^*,#^
tHCY (µmol/L)	6.6 (6.0 – 7.8)	7.6 (5.6 – 9.8)	7.9 (6.6 – 9.5)^*^
Cysteine (µmol/L)	200 (190 – 220)	200 (190 – 250)	220 (202 – 250)^*^
Methionine (µmol/L)	86 (55 – 98)	83 (62 – 106)	70 (66 – 99)
**B12, Cbi and creatinine in urine^3^**			
u–B12/u–Crea (nmol/mmol)	3 (3 – 4)	18 (10 – 29)**	14 (6 – 85)×10^3 ***,###^
u–Cbi/u–Crea (nmol/mmol)	1 (1 – 6)	14^.^ (7 – 21) x^.^10^3**^	N.D.^4^
u–MMA/u–Crea (μmol/mmol)	15 (10 – 28)	20 (14 – 30)	20 (13 – 29)
u–crea (µmol/24 h)	1.7 (1.3 – 2.4)	1.1 (0.6 – 1.6)^*^	1.1 (1.0 – 1.9)
**B12 and Cbi in tissues**			
Kidney–B12 (pmol/g)	1800 (1500 – 2100)	590 (450 – 710)^***^	6900 (6000 – 9400)^***;###^
Kidney–Cbi (pmol/g)	<50	2700 (1400 – 2900)^ ***;###^	<50
Liver–B12 (pmol/g)	320 (290 – 390)	170 (140 – 200)^***^	530 (390 – 610)^***;###^
Liver–Cbi (pmol/g)	<2	690 (630 – 920)^***;###^	<2
Salivary gland–B12 (pmol/g)	130 (110 – 160)	59 (18 –96)^***^	180 (150 – 290)^ *;###^
Salivary gland–Cbi (pmol/g)	<10	530 (240 – 920)^***;###^	<10
Salivary gland–UB12BC (pmol/g)	79 (33 – 180)	10 (1.2 – 16)^*^	2.8 (1.0 – 16)^*^

1: The listed values are obtained from samples collected from mice 27 days after subcutaneous insertion of an osmotic minipump delivering 0.9% NaCl (control), 4.25 nmol/h Cbi, or 1.75 nmol/h B12 in 7–week–old female mice of the 129.S6 strain. n = 7 in each group.

2: ANOVA tests are carried out between Control and Cbi– or B12–mice (*p<0.05; **p<0.001; ***p<0.0001) as well as between Cbi and B12 mice (#p<0.05; ##p<0.001; ###p<0.0001). For values below analytical detection limit, the detection limit has been used in the calculations.

3: n = 6 for urine values of control mice.

4: The used assay for analysis of Cbi in urine does not allow for determination of small amounts of Cbi in B12–rich samples.

ND: Not determined. tHcy: total homocysteine. UB12BC: Unsaturated B12 binding capacity. B12: Vitamin B12 Cbi: cobinamide. Crea: creatinine. MMA: methylmalonic acid.

Although mice TC is able to bind analogues *in vitro* [2], the level of B12 analogues was below the detection limits in plasma and tissue samples of the control mice. This was surprising as up to 50% of the total load of corrinoids (B12 and B12-like compounds) found in human plasma are B12 analogues [15].

**Figure 2:**
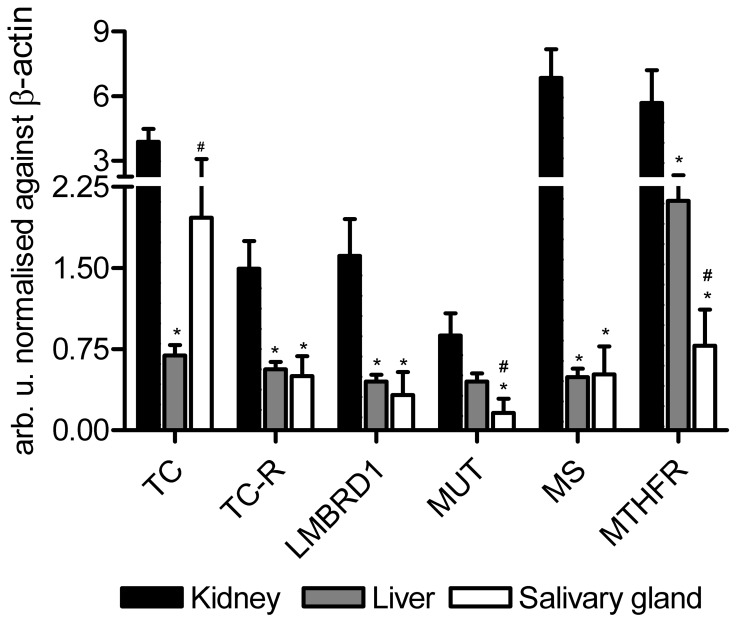
Transcript levels of selected genes involved in B12 metabolism in mice. The columns shown mean (+SEM) mRNA levels determined by quantitative reverse–transcriptase (q–rt–) PCR and normalised against β–actin in kidney, liver, and salivary glands of control mice (n = 7 mice of each tissue). Arb.u.: Arbitrary units. LMBRD1: lysosomal B12 transporter. MS: methionine synthase. MTHFR: methylenetetrahydrofolate reductase. MUT: methylmalonylCoA mutase. TC: transcobalamin. TC–R: TC–receptor. Wilcoxon tests were carried out between kidney and liver or salivary glands (*p<0.05) as well as between liver and salivary glands (#p<0.05).

The transcript levels measured by q-rt-PCR of genes encoding key proteins in the cellular uptake and turnover of B12 are shown in Figure 2. The highest transcription levels were observed in the kidney. In accord with previous data, TC is highly transcribed, also in the salivary glands [2].

### Osmotic minipumps deliver a continuous load of Cbi or B12

Initial experiments showed that subcutaneous injection with Cbi or B12 resulted in a significant, but short-term increase in plasma levels of the compounds (results not shown). Using osmotic minipumps inserted subcutaneously, we secured a continuous load of Cbi, B12, or vehicle alone (0.9% NaCl). Due to differences in solubility [17], Cbi mice received 4.25 nmol/h Cbi, and B12 mice received 1.75 nmol/h B12.

To ensure continued delivery of B12 or Cbi, we measured unsaturated B12 binding capacity (UB12BC) in plasma and 24-h-urinary excretion of B12 prior to and then weekly during the 27-day infusion period. The initial values did not differ between the three groups (not shown). UB12BC in plasma remained constant in the control mice (∼28 nM) throughout the study. In both Cbi- and B12-loaded mice, UB12BC was below the detection limit from the first measurement 6 days after inserting the pumps (data not shown) and remained so to the day of sacrifice (Table 2). Together, these results show that the mice were steadily loaded with Cbi and B12 at least from day 6 and throughout the remaining part of the study. In B12-loaded mice, urinary B12 secretion corresponded to 25–33% of the calculated infused amount of B12 (42 nmol/24 h). The urinary excretion of Cbi corresponded to 10–17% of the calculated Cbi load (102 nmol/24 h).

### Continuous delivery of Cbi or B12 has limited influence on plasma markers

Loading with Cbi or B12 resulted in a two-fold increase in white blood cell count in the B12-treated mice and a slight, although not statistical significant increase in the Cbi-treated mice. Apart from this, we did not observe major changes in haematological parameters related to B12 deficiency (Table 2).

The level of MMA, tHCY, cysteine, and methionine remained unchanged in Cbi-loaded animals, suggesting that these animals did not suffer from overt B12 deficiency at the time of sacrifice. The B12-loaded animals showed a decrease in MMA level. Also, a small but significant increase in both tHCY and cysteine was seen. The plasma level of B12 increased to more than 100 nM in B12-loaded animals, and the plasma level of B12 analogues was high in Cbi-loaded animals, reaching levels of more than 600 nM, while the level of B12 showed a slight but significant decrease in these mice (Table 2).

### Continuous delivery of Cbi or B12 alters tissue B12 levels

We observed significant alterations in the tissue content of B12 in mice treated with Cbi or B12. In Cbi-treated animals, kidney B12 concentrations were reduced to approximately 33% of that in control animals, while concentrations in the liver and salivary glands were approximately 50% of the controls. We detected substantial amounts of Cbi in all tissues from animals treated with this compound. The B12-treated mice showed B12 levels in the kidney that were around 4 times higher those seen in the control mice, whereas increases in the liver and salivary gland were less pronounced.

### Continuous delivery of Cbi or B12 alters transcript levels differently between tissues

**Figure 3:**
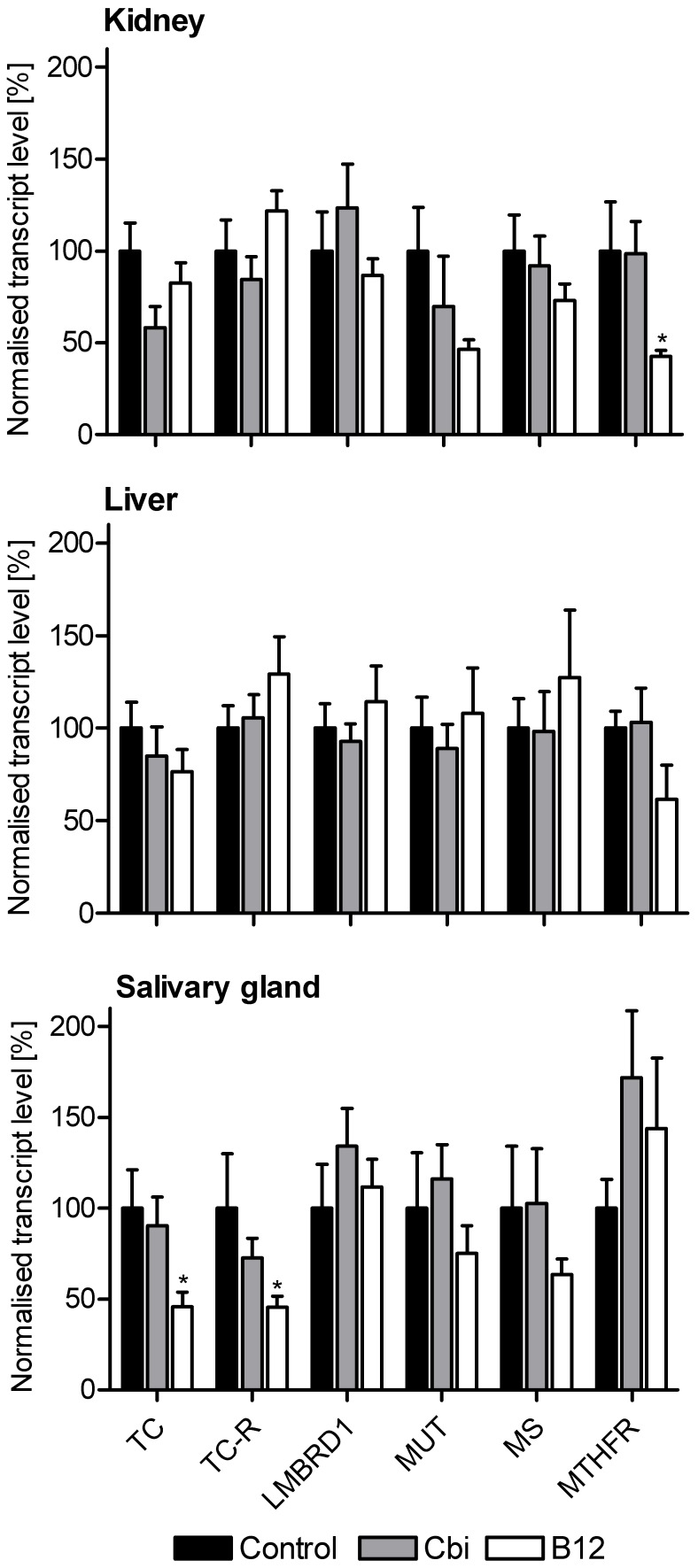
Changes in transcript levels after treatment of mice with Cbi or B12. Kidney, liver and salivary glands were removed from mice treated for 27 days with saline (control, n = 7), 4.25 nmol/h Cobinamide (Cbi, n = 7) or 1.75 nmol/h vitamin B12 (B12, n = 7). We analysed the transcript levels by q–rt–PCR. The results (Mean +SEM) for each sample are relative to the mean value for the corresponding tissue from control mice. Non–parametric Wilcoxon tests were employed to calculate significant differences, p<0.05 are indicated with * for comparison to control mice. No significant difference was observed between Cbi and controls or Cbi and B12 mice. LMBRD1: lysosomal B12 transporter. MS: methionine synthase. MTHFR: methylenetetrahydrofolate reductase. MUT: methylmalonyl–CoA mutase. TC: transcobalamin. TC–R: transcobalamin receptor (CD320).

No significant changes in the transcript levels of TC-R, LMBRD1, MUT, MS, and MTHFR were observed following Cbi loading in any of the analysed tissues (Figure 3). In kidneys of B12-loaded mice, the transcript level of MTHFR was reduced both compared to Cbi and to control mice. In salivary glands of B12-treated mice, transcript levels of TC and TC-R were reduced to 50% compared to both control and Cbi mice. This picture indicates that only B12 loading influences transcript levels and that the changes are tissue specific.

## Discussion

We present a mouse model that allows loading of compounds delivered via the B12 transport system and show data to indicate that loading with both active B12 and an inactive compound leads to changes in B12 accumulation and metabolism.

We recently showed that mouse TC, the only B12 carrier in mouse plasma, binds both B12 and the inactive B12 analogue Cbi *in vitro* [2], although the binding affinity for Cbi is around 10-fold less than the binding affinity for B12. Based on this observation, we designed a mouse model that allowed continuous saturation of the endogenous mouse TC by Cbi (4.25 nmol/h) or B12 (1.75 nmol/h) by administration through osmotic minipumps inserted subcutaneously.

All circulating TC remained saturated with Cbi or B12, and excess Cbi and B12 was steadily excreted in the urine from day 6 and throughout the 4-week experiment. Combined, these observations indicate that the transport system was maximally loaded at least from day 6 and onwards, thus providing a proof of concept in relation to the model.

Pharmacological B12 conjugates are designed to use the B12 uptake and distribution system. Because of that, they will compete with the endogenous B12 for the association to B12 binding proteins, not only in the digestive system (binding to intrinsic factor and uptake of intrinsic factor B12 complexes (for review see [1]), but also in the blood and cells (binding to carrier proteins and receptors) (See Figure 1). In the presented model we have examined the capacity for uptake through the B12 transport system and also evaluated possible effects of a load with either B12 or its analogue Cbi. As very high doses of B12 and Cbi were administered, we cannot exclude passive cellular uptake of unbound B12.

We have recently shown that kidney, liver, and salivary gland are the mouse tissues that show the highest content of B12, TC, and/or TC-R [2]. Therefore, we focused our analyses on the changes in the B12 level and markers of B12 metabolism in these organs.

### Capacity for delivery of compounds through the B12 transport system

In mice, half the amount of circulating transcobalamin is unsaturated with B12 (Table 2). Thus, if TC was the only limiting factor for uptake of the vitamin, one would expect that tissue Cbi/B12 would amount to around twice the B12 level observed in control animals. While B12 accumulation in liver and salivary glands of B12-treated mice approached a factor of 2 and 1.5, respectively, accumulation in the kidney far exceeded a factor of 2. This is explained by the kidney's role in rodent B12 metabolism. The kidney acts as a reservoir for B12 and accumulates the vitamin during loading, whereas the vitamin is liberated from the kidney during deficiency [18].

The results for the Cbi-loaded animals show cellular depletion of B12 and a substantial accumulation of Cbi, notably in the liver and salivary glands, where the sum of B12 and Cbi far exceeds a factor of 2 as compared to the level of B12 in control mice. The accumulation of Cbi may in part be caused by passive uptake in the cells. Another possibility is that the export of Cbi from the cells is slower than is the export of B12. The kidney displayed another pattern. B12 was markedly depleted, and accumulation of Cbi was relatively low (Table 2). This observation may suggest that Cbi is liberated from the kidney in parallel to liberation of B12 for the rescue of B12 depletion in other tissues of the body.

### High-dose Cbi depletes tissue B12 but does not influence markers of B12 metabolism

The plasma level of B12 decreased approximately 30% after treatment with Cbi, but within the study period, we did not observe any clinical changes comparable to those seen in humans lacking B12. In addition the red blood cell counts were unchanged and so were the levels of the two metabolic markers of vitamin B12 deficiency tHCY and MMA (Table 2). However, little is known about the sensitivity of these biomarkers in mice; notably, they have previously been shown to remain stable in knock-out mice with reduced tissue B12 levels [19]. In both treated groups, the white blood cell count was increased and to the highest level in the B12 treated mice. A similar response has been observed in excessive B12 load of healthy humans [20]. In that study, it was explained that B12 acts as cellular modulator in the immune response system. We have no specific explanation to offer for this observation and it significance remains to be established.

In Cbi-loaded mice, Cbi was internalised into the tissues at the expense of B12, as B12 levels decreased in the kidney (3-fold), liver (2-fold), and salivary glands (2-fold). In addition, the Cbi-treated mice excreted more B12 in the urine than did control animals. Together, we take these results to indicate an active transport of Cbi into the cells in competition with endogenous B12. Cbi treatment did not affect gene transcription for any of the genes studied. We interpret these results to support the notion that the mice do not develop major changes in their B12 metabolism even after a reduction of tissue B12 levels to 33–50% of those observed in control mice. However, this study only lasted 4 weeks. Prolonged treatment with an inactive B12 analogue such as Cbi could further deplete B12 from the cells and, in time, cause B12 deficiency.

### High-dose B12 increases tissue B12 and influences markers of B12 metabolism

High doses of B12 increased plasma B12 and decreased the level of MMA. Unexpectedly, the other metabolic marker of B12 deficiency, tHCY, increased and so did cysteine, another metabolite of the homocysteine pathway. To our knowledge no previous studies have shown that B12 load may lead to an increased level of tHCY, thus suggesting that in relation to the metabolism of homocysteine, both too little B12 and too much may be disadvantageous.

Our results show that the major part of surplus B12, and therefore possibly also B12 conjugates, will end up in the kidney. The liver is able to double the amount of B12 internalised.

High dose B12 resulted in previously undescribed changes in the transcript levels of genes involved in the transport and metabolism of the vitamin. Firstly, we found a reduced transcript level of B12-dependent enzyme MTHFR in the kidneys. This reduction was not related to an accumulation of methionine in this group of mice. This could be expected, as MTHFR is involved in the conversion of methionine into methyl-tetrahydrofolate, see Figure 1. but apparently, the reduction in MTHFR mRNA was not enough to cause accumulation of methionine. Secondly, we found that TC and TC-R are reduced in salivary gland. We have no explanation to offer for this observation. However, a down-regulation of the TC-R fits well with our observation of a relatively low accumulation of salivary gland B12 in the B12-treated mice.

### Implications for future studies using B12 conjugates as pharmacological treatments

Our studies have several implications. We show that in mice, the TC-mediated B12 transport system has an excess capacity that may be used for the transport of B12 conjugates. The capacity for uptake of B12 conjugates in humans may be even larger since in humans only around 10% of the circulating TC is saturated with B12 [21], while in mice, the saturation degree is 50% (Table 2). Excess B12 is not evenly distributed in the mouse tissues, which has also been observed in humans [22]. Thus, the use of the B12 transport system for the delivery of drugs may prove most efficient if targeted to tissues that accumulate the vitamin such as the kidney and the liver. After B12 loading, alterations in the expression of B12-related genes and circulating tHCY were observed. This may have implications for treatment with B12 conjugates that dissociate to active B12 and conjugate within the cell. Our study also indicates that B12 conjugates compete with the transport of B12 into the cells. Thus, if the conjugate is inactive with respect to B12, this may lead to changes in B12 status.

### Conclusions

Taken together, our study shows that mice are a suitable model for studies of the transport and effect of B12 conjugates. Furthermore, our study emphasises the importance of monitoring B12 metabolism during treatment with B12 conjugates or analogues.
